# Low Maternal Vitamin D Status during the Second Trimester of Pregnancy: A Cross-Sectional Study in Wuxi, China

**DOI:** 10.1371/journal.pone.0117748

**Published:** 2015-02-06

**Authors:** Jian-Ping Xiao, Jia Zang, Jing-Jing Pei, Fei Xu, Yan Zhu, Xiang-Peng Liao

**Affiliations:** 1 Department of Obstetrics, Wuxi Maternity and Child Health Hospital, Nanjing Medical University, Jiangsu, China; 2 Central Laboratory, Wuxi Maternity and Child Health Hospital, Nanjing Medical University, Jiangsu, China; 3 Department of Health Care, Wuxi Maternity and Child Health Hospital, Nanjing Medical University, Jiangsu, China; 4 Department of Newborn, Wuxi Maternity and Child Health Hospital, Nanjing Medical University, Jiangsu, China; 5 Perinatal Biology Center, Soochow University School of Medicine, Jiangsu, China; 6 Department of Obstetrics and Gynecology, Sherbrooke University Hospital Center, Sherbrooke, Canada

## Abstract

**Background:**

Vitamin D deficiency is common in pregnant women, but an optimal serum vitamin D level during pregnancy has not been determined and remains an area of active research. Vitamin D data from large populations of pregnant Chinese women are still limited.

**Objective:**

To evaluate the vitamin D status of women in Eastern China during the second trimester of pregnancy.

**Methods:**

A hospital-based, cross-sectional, observational study. Serum 25-hydroxyvitamin D [25(OH)D] concentration was measured in samples from 5823 pregnant women in Wuxi City, China (latitude: 31.5o N), from January 2011 to June 2012.

**Results:**

The median serum 25(OH)D concentration was 34.0 nmol/L [2.5 nmol/L 25(OH)D = 1 ng/mL 25(OH)D]. Vitamin D deficiency [defined as 25(OH)D < 30 nmol/L according to the Institute of Medicine (National Academy of Sciences, Washington, D.C., USA)] or inadequacy [25(OH)D of 30–49.9 nmol/L] was identified in 40.7% and 38.0% of the women, respectively. Only 0.9% had a 25(OH)D level ≥ 80.0 nmol/L, which is the concentration recommended as adequate by the Endocrine Society (Washington, D.C., USA). Compared with older women, younger women were more likely to be deficient in vitamin D. There were significant differences in the 25(OH)D levels according to season. The 25(OH)D levels reached peak values in September and were correlated with (r = 0.337, P < 0.001), and fluctuated with, average monthly air temperatures.

**Conclusions:**

There is a high prevalence of Vitamin D deficiency among pregnant Chinese women, and 25(OH)D levels varied according to season and air temperature. The results of this study also suggest that currently there is a big gap between the levels of Vitamin D detected in pregnant Chinese women and the levels recommended by the Endocrine Society.

## Introduction

There has been increasing interest in the roles of vitamin D in human health. Relationships between vitamin D intake and health outcomes range from effects on bone health to the potential role of vitamin D for the prevention of nonskeletal disorders (e.g., auto-immune disease, cancer, mental health disorders, and cardiovascular disease) [1–4]. Vitamin D may also have a role in the prevention of diabetes and preeclampsia during pregnancy [5].

Complete agreement on the cut-point values for 25-hydroxyvitamin D [25(OH)D] concentrations that should be used has not been reached. The Institute of Medicine (IOM; National Academy of Sciences, Washington, DC, USA) and the National Osteoporosis Society (Bath, England) agree on the following serum 25(OH)D levels for bone health [2.5 nmol/L 25(OH)D = 1 ng/mL 25(OH)D]: < 30 nmol/L indicates deficiency, 30–49.9 nmol/L may be inadequate in some people, and ≥ 50 nmol/L is adequate for most of the population [3,4]. However, the Endocrine Society (Washington, D.C., USA) defines serum 25(OH)D concentrations ≤ 75–80 nmol/L as inadequate [2]. The American College of Obstetricians and Gynecologists (ACOG) states that an optimal serum vitamin D level during pregnancy has not been determined, that the subject remains an area of active research, and that there is as yet insufficient evidence to recommend routine screening for vitamin D deficiency in all pregnant women [6]. Regarding to the Recommended Dietary Intake of vitamin D for pregnant women, the Institute of Medicine established recommends 600 international units per day (IU/D, 1 IU = 25 ng) [4], while the Endocrine Society suggests the amount of 600–2000 IU/D as Daily Requirement for pregnant woman, who might be at risk of vitamin D deficiency [2]. In China, pregnant women rarely take vitamin D supplements during the first and second trimesters of pregnancy. Many obstetricians prescribe calcium supplements starting in the second or third trimester of pregnancy. Certain of these supplements contain small concentrations of vitamin D (approximately 100–200 IU/D). There is increasing evidence indicating that maternal vitamin D deficiency during pregnancy is common [5,7–9]. However, vitamin D data from large populations of pregnant Chinese women are still limited. The objective of this study was to evaluate the vitamin D status of pregnant women using a large sample size, and to analyze related factors.

## Materials and Methods

### Ethics statement

This was a hospital-based, non-interventional, cross-sectional, observational study. The Medical Research Ethics Board of Wuxi Maternity and Child Health Hospital affiliated with Nanjing Medical University approved the study. The pregnant women were recruited monthly on the basis of convenience, and informed about the research and its objectives. After written consent to participate was obtained, we measured the serum 25(OH)D level. The study was approved by the Hospital Research Ethics Board.

### Data collection

The study was implemented at a hospital affiliated with Nanjing Medical University. The hospital is located in Wuxi city in Eastern China’s Jiangsu Province, at latitude 31.5^°^ N, and is approximately 120 kilometers from Shanghai. The investigation period was from January 2011 to June 2012. Wuxi has an urban population of about 3.6 million individuals. The hospital mainly provides service for these urban residents, and performs approximately 12,000 deliveries per year.

Pregnant women (Han nationality) aged 18 to 35 years who had lived at their urban locations for > 1 year and had participated in the prenatal care program at the Hospital Fetal Medicine Center from approximately 8 weeks of gestation from their last menstrual periods were invited to participate in the study. Women with the following criteria were excluded from the study: 1) those with chronic metabolic diseases impacting vitamin D metabolism, such as hypothyroidism, hyperthyroidism, liver and kidney diseases; 2) those with mental illnesses; 3) pregnant women and their spouses with a family history of the following diseases: mental retardation, genetic metabolic diseases, serious congenital malformations; 4) pregnant women or their spouses addicted to substances such as tobacco, alcohol or drugs; 5) those withholding consent to participate.

Gestational age was calculated from the mother’s last menstrual period and was confirmed by obstetric ultrasound. Year-round data for air temperature were obtained from the Bureau of Meteorology, Wuxi City.

### Estimating the concentrations of 25(OH)D

The blood samples for 25(OH)D measurement were collected and stored at -40°C. The samples were taken at 15–18 weeks of gestation (i.e., during the second trimester) at the same time that maternal blood was drawn for the screening tests of fetal chromosomal disorders. Serum 25(OH)D concentrations were measured using enzyme-linked immunosorbent assay (ELISA) (IDS Diagnostics Ltd, Boldon, Tyne & Wear, UK). The intra- and inter-assay coefficients of variation were < 10% respectively.

### Statistical analysis

The pregnant women were classified into three age groups (18–24 years, 25–29 years, and 30–35 years) using the age classification of regional patient information and statistics. We followed Institute of Medicine guidelines to establish the cutoffs for vitamin D levels [3,4]. Vitamin D deficiency was 25(OH)D < 30 nmol/L, vitamin D inadequacy was 25(OH)D = 30–49.9 nmol/L, and adequate levels were 25(OH)D ≥ 50 nmol/L. The seasons for sample collection were defined as spring (March, April, May), summer (June, July, August), autumn (September, October, November), and winter (December, January, February).

The statistical analysis was performed using SPSS 21.0 (IBM Corp, Armonk, NY, USA). Analyses of the data for the serum 25(OH)D concentrations revealed the presence of a skewed distribution. The data were therefore described using median and percentile values. The non-parametric chi-square test was used to compare differences between groups. Pearson correlation analysis was used to estimate partial correlation coefficients. The results were considered to be statistically significant when the 2-tailed *P*-value was < 0.05.

## Results

**Fig 1:**
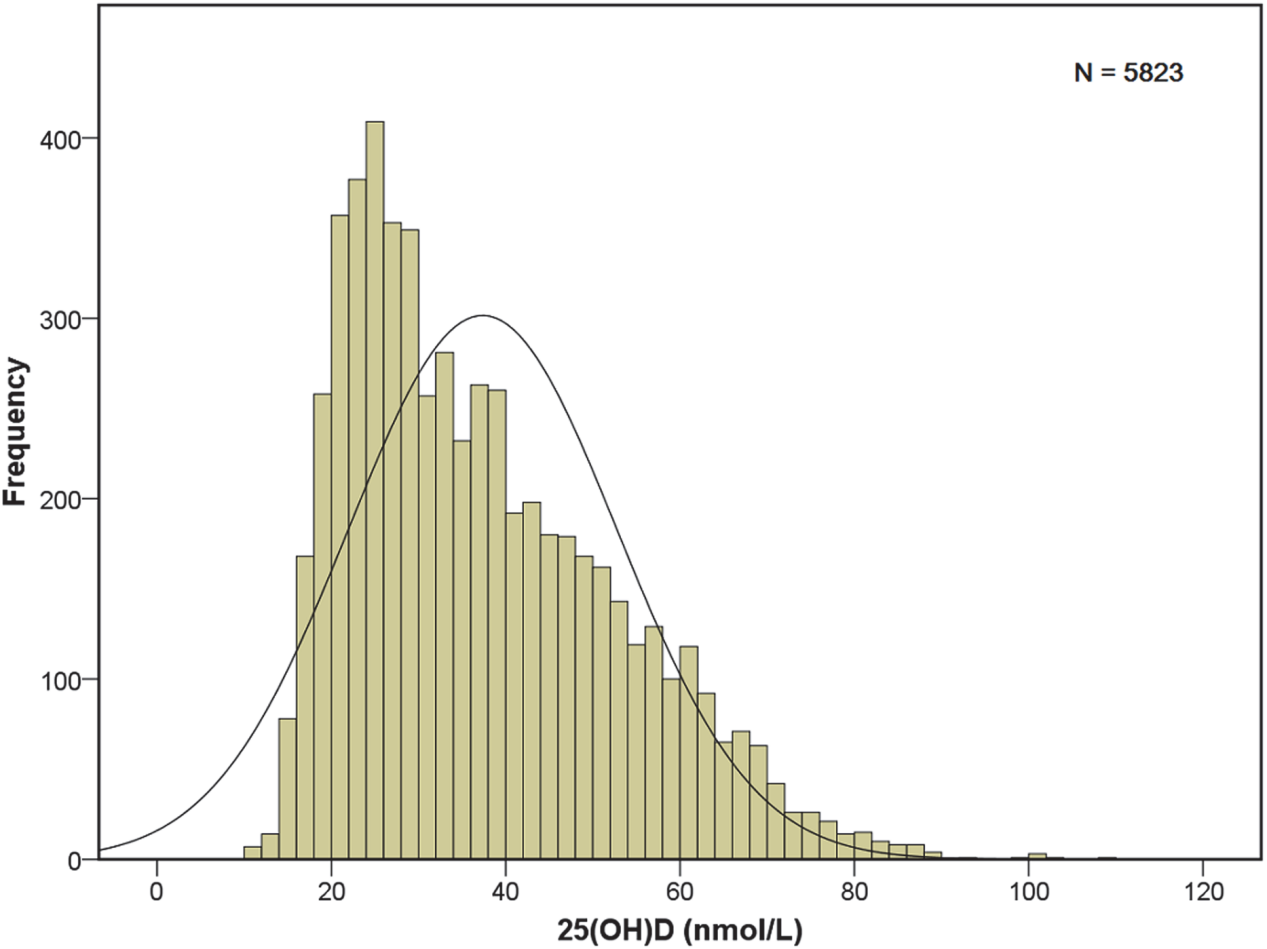
Frequency distribution of the 25(OH)D measurements. 25(OH)D concentration is shown as a skewed distribution, and most of the values are less than 30 nmol/L.

A total of 5823 urban pregnant women were recruited for this investigation. The mean age of the participants was 26.4 ± 3.1 years (range, 18–35 years). The frequency distribution for the 25(OH)D concentrations was skewed to the left, and most of the results were < 34 nmol/L (Fig. 1).

**Fig 2:**
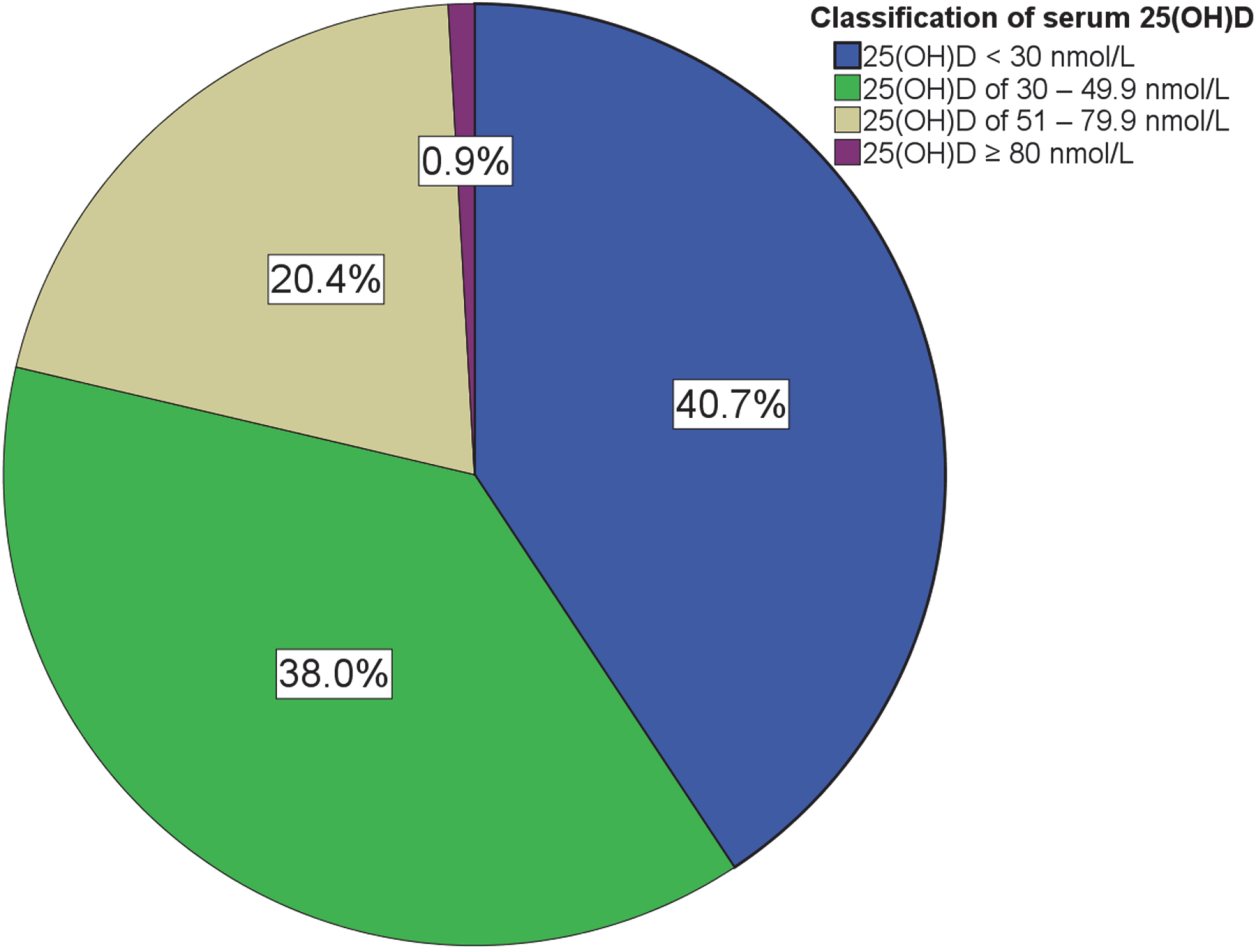
Proportions of different vitamin D categories in the study population.

The median serum 25(OH)D concentration was 34.0 nmol/L (range, 10.1–108.7 nmol/L). Vitamin D deficiency and inadequacy were present in 40.7% and 38.0% of the women, respectively. Only 0.9% of the study population had 25(OH)D concentrations ≥ 80.0 nmol/L. During the winter season, most (57.3%) of the women were deficient in vitamin D, and only 6.8% of them had adequate vitamin D levels (Table 1 and Fig. 2).

**Table 1:** Levels and percentages of 25(OH)D measurements in pregnant women, among season and age group.

Group	N	Median	Percentile (p25—p75)	Deficient (%)	Inadequate (%)	Adequate (%)
		(nmol/L)	(nmol/L)	(< 30 nmol/L)	(30–49.9 nmol/L)	(≥ 50 nmol/L)
**Season** *						
Spring	2079	33.8	(24.5–46.2)	41.0	39.8	19.2
Summer	1077	44.0	(29.3–57.2)	26.6	34.1	39.3
Autumn	1085	39.6	(26.9–47.3)	29.9	41.4	28.8
Winter	1582	28.2	(23.0–36.4)	57.3	35.9	6.8
**Age** **						
18–24 years	2533	32.8	(24.5–46.4)	42.4	38.3	19.3
25–29 years	2701	35.6	(25.2–50.0)	40.2	37.2	22.6
30–35 years	589	36.2	(25.2–48.2)	35.8	40.1	24.1
Total	5823	34.0	(25.0–47.4)	40.7	38.0	21.3

* χ^2^ = 470.75, *P*<0.001. The vitamin D levels were significantly different among different season groups.

** χ^2^ = 1419.87, *P*<0.001. The vitamin D levels were significantly different among different age groups.

**Fig 3:**
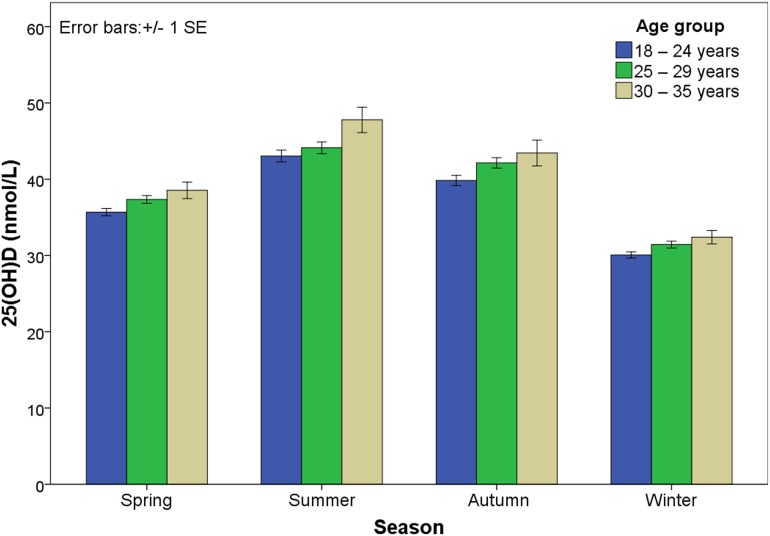
Histogram of 25(OH)D measurements classified by season and age group. The 25(OH)D levels in pregnant women were highest in the summer season and the lowest in the winter season. For different seasons, the 25(OH)D level in the 30–35 year age group was higher than that in 18–24 and 25–29 year age groups.

Vitamin D levels were significantly different according to seasons (χ^2^ = 470.75, *P* < 0.001). The lowest vitamin D levels occurred during the winter season, and the highest levels occurred during the summer season. Vitamin D levels were significantly different among different age groups (χ^2^ = 1419.87, *P <* 0.001). The vitamin D levels in the 30–35 year age group were higher than those in the 25–29 year and the 18–24 year age groups (Fig. 3 and Table 1).

The 25(OH)D concentration was correlated with air temperature (*r* = 0.337, *P* < 0.001) for all subjects. The correlation coefficients between the 25(OH)D values and air temperature for different age groups were calculated. The coefficients were approximately similar (0.327, 0.348, and 0.358 for the 18–24, 25–29, and 30–35 year age groups, respectively, all *P* < 0.001).

**Fig 4:**
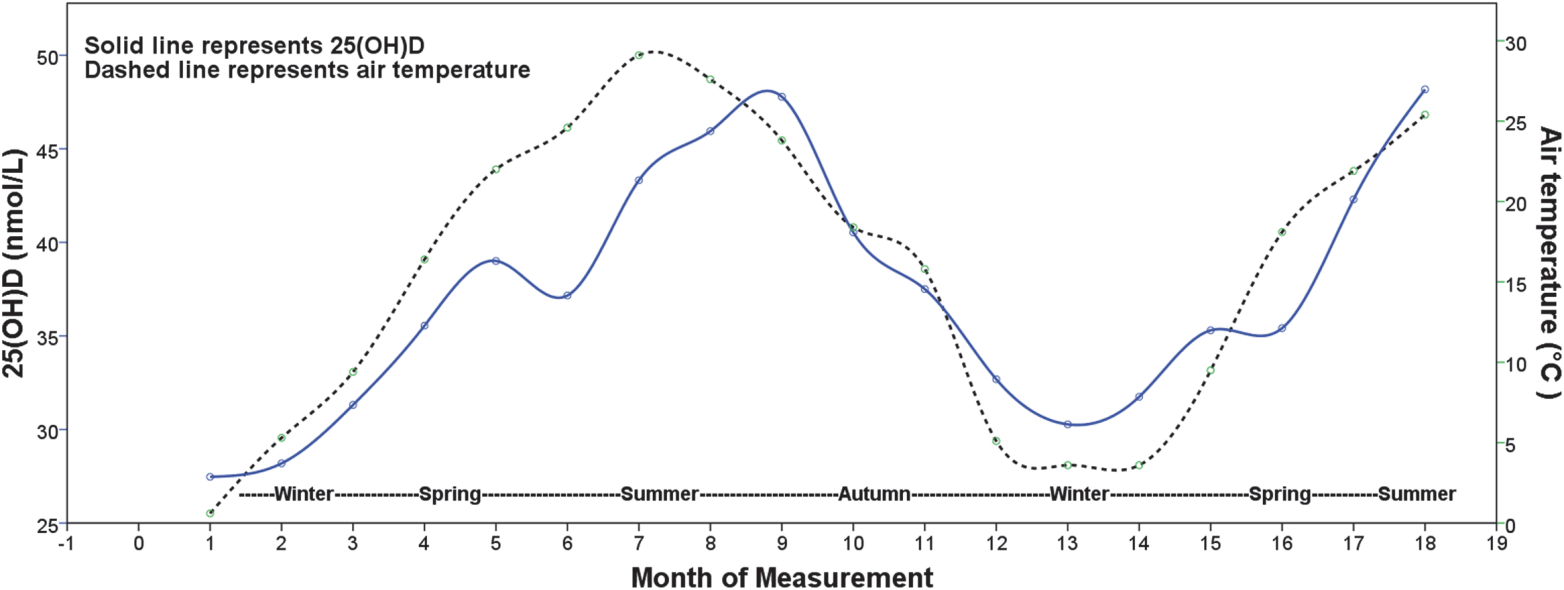
Trends in 25(OH)D results and in air temperature variation. From January 2011 to June 2012, the 25(OH)D levels and average monthly air temperature values showed regular variation and the shapes of the curves were consistent with a sinusoidal pattern.

Fig. 4 presents the results for trends in the 25(OH)D and average monthly air temperature values, by month and season of measurement. The 25(OH)D levels and air temperature values showed regular, sinusoidal, variation. The lowest vitamin D levels and air temperatures occurred in January. The changes in 25(OH)D levels were fairly consistent with the changes in air temperature. The peak value in air temperature occurred in July, and then declined. The 25(OH)D concentrations were highest in September, and then declined to their lowest values, which occurred in January. Values for both variables then began another cycle.

## Discussion

Several reports on vitamin D status across different regions and populations have been published in recent years. We used clinical data with a large sample size and found that there was a high prevalence of vitamin D deficiency in pregnant women in China. We also found that the 25(OH)D levels in pregnant women were correlated with air temperature. The levels fluctuated with air temperature and had an analogous trend.

Our data show that maternal vitamin D deficiency is prevalent in China as well as in Western countries. We used the cut-points recommended by the Institute of Medicine and found that 40.7% of urban pregnant women were vitamin D deficient. Only 21.3% had adequate levels of vitamin D. The results of a study from China indicated that 69% of urban pregnant women have a 25(OH)D level < 50 nmol/L [10]. During the winter and spring seasons, more than 50% of school age children and adolescents have a 25(OH)D level < 50 nmol/L [11]. However, an analysis of data from the USA revealed that only an estimated 5% to 29% of pregnant American women may have inadequate vitamin D status [defined as 25(OH)D < 37.5 nmol/L] [12,13], and 7% of American pregnant or lactating women are at risk for vitamin D deficiency [25(OH)D < 30 nmol/L]. Results from a national survey indicated that an additional 21% are at risk for vitamin D inadequacy [25(OH)D < 50 nmol/L] [14]. Meanwhile, vitamin D levels recorded from pregnant women in recent cohort studies in other Western countries have been higher compared with levels in pregnant Chinese women. For example, for 25(OH)D values in the UK, overall median values of 65 nmol/L [15], and 55, 60, and 67 nmol/L have been recorded for the first, second, and third trimesters, respectively [16]. A median value of 53 nmol/L has been reported for Belgian women [9], a mean value of 57 nmol/L has been reported for Caucasian women in Canada [17], and a mean value of 57 nmol/L has been reported for pregnant women in Australia [18].

An optimal serum 25(OH)D level during pregnancy has not been determined. Only 0.9% of all the women in this study had a level of 25(OH)D ≥ 80 nmol/L (defined as adequate by the Endocrine Society). To some degree, these results suggest that pregnant Chinese women may not really require the higher vitamin D levels recommended by the Endocrine Society. There are no results from our study for other biochemical markers to support this hypothesis. However, results of other reports from China also indicated that vitamin D levels are lower, and that there are different relationships between vitamin D and parathyroid hormone (PTH) levels. In the mid-1990s, none of 1248 girls aged 12–14 years in Beijing (latitude 39.9°N) had 25(OH)D levels > 70 nmol/L [19]. Only 3.9% of pregnant women living in the sunny city of Guiyang, China (latitude 26.6°N) had adequate levels of vitamin D [25(OH)D ≥ 80 nmol/L] [20]. Over 90% of women in Beijing and Hong Kong have been reported to have inadequate levels of vitamin D [25(OH)D < 50 nmol/L], and there was a linear relationship between 25(OH)D and PTH concentrations, with no apparent threshold [21].

Low serum 25(OH)D concentrations are more common and more severe among racial/ethnic minorities in Western countries. In these populations, the distributions of 25(OH)D concentration, and other aspects of 25(OH)D metabolism, might differ [22,23]. Vitamin D may not affect health in similar ways in different racial/ethnic groups. There may be differences in its effects on coronary heart disease events [24], biomarkers, and bone mass [23,25]. Taken together, these results suggest that current vitamin D cut-points recommended for vitamin D standards by the Endocrine Society may not be applicable or appropriate for all racial groups.

It has been known that the circulating 25(OH)D is regularly influenced by season [26,27]. To our knowledge, we have firstly found that 25(OH)D levels are correlated with (*r* = 0.337), and fluctuated with, air temperature. The effect of air temperature on vitamin D status is via exposure of skin to ultraviolet B radiation. For most people, the majority (80–90%) of circulating 25(OH)D is produced by the action of ultraviolet B radiation on skin 7-dehydrocholesterol [3]. Results from the city of Nanjing (170 kilometers from Wuxi city) indicate that the seasonal variations in ultraviolet radiation intensity fluctuate in a sinusoidal pattern [28]. These variations in ultraviolet radiation intensity are visually the same as the variations in the air temperature in our results. This correspondence might provide an explanation for the seasonal variations in 25(OH)D that we found our investigation. Ultraviolet radiation intensity is also influenced by rain, and by cloud cover. In the Yangtze River Delta region of Eastern China, rainy weather and cloud cover prevail during the spring and summer seasons. These patterns in environmental conditions might explain the small irregular fluctuations in 25(OH)D levels.

We also found that the vitamin D levels in younger pregnant women (18–24 years) were lower than they were in the older pregnant women (30–35 years). The correlation coefficient with air temperature was slightly lower in younger pregnant women (*r* = 0. 327) compared with the older pregnant women (*r* = 0. 358). Older pregnant women might have greater exposure to sunlight and be more likely to comply with prenatal care recommendations, such as calcium supplementation, which might simultaneously contain vitamin D, while the separate vitamin D supplements during pregnancy is currently an infrequent clinical practice in China. This higher prevalence of vitamin D deficiency and insufficiency among younger pregnant women was also found in younger pregnant women in the USA [7].

The potential limitations of this study include the lack of detailed demographic and morphological characteristics for all of the subjects. Some data (e.g., nutritional intake, outdoor activities, lifestyle, and maternal and fetal outcomes) were recently collected from a small subgroup of our study cohort, but have not yet been analyzed. To our knowledge, this report represents the largest sample size survey of vitamin D status in pregnant Chinese women. The results provide a compelling basis for further research.

In summary, we found that there was a high prevalence of vitamin D deficiency and inadequacy in pregnant Chinese women, which varies according to season and air temperature. With this study we demonstrate a relevant difference between the levels of 25(OH)D in pregnant women in Wuxi, China and the levels recommended by the Endocrine Society. Strategies to optimize vitamin D status in pregnant women should therefore be developed.
